# Outcome measures in systemic lupus erythematosus: constructing a meaningful response index from existing clinical trial data

**DOI:** 10.1186/ar4659

**Published:** 2014-09-18

**Authors:** Lindsy Forbess, Daniel Wallace, Mariko Ishimori, Michael Weisman

**Affiliations:** 1 Cedars-Sinai Medical Center, Los Angeles, CA, USA

## Background

The purpose of this project is to develop a systemic lupus erythematosus (SLE) response index as a standard outcome measure in future therapeutic trials. Currently, there is no widely validated method for defining response to therapy. Most SLE trials to date have failed to meet predesigned endpoints, leading to controversy over whether it is drug treatments or outcome measures that are unsuccessful in SLE. A similar controversy in rheumatoid arthritis (RA) years ago was resolved by examining data from placebo-controlled trials with drugs that were only modestly effective. Important clinical variables were selected, criteria for patient improvement determined, and an index was developed that distinguished treated patients from those getting placebo. This index (ACR 20/50/70) used in RA trials has led to approval of more than 20 drug therapies.

## Methods

Now that large-scale SLE clinical trial data exist, we propose to use the approach that was successful in RA. We will perform a *post-hoc *analysis of the raw data from the BLISS-52 and BLISS-76 trials investigating belimumab for SLE. The disease activity indices (SELENA SLEDAI and BILAG) will be deconstructed and individual clinical and laboratory parameters will be identified (for example, rash, complement). The variables that are present in the majority of patients, improve over time, and have face validity will be selected for this index. Both the physician global assessment and a patient-related measure of quality of life will be included.

## Results

Study data will be split 50/50 into a training set and a validation set. Baseline values of variables will be compared with values at the end of the study to determine the degree of improvement or deterioration occurring in individual patients during the study. We will examine various threshold percent-improvement cutoff points across sets of variables, selecting those that produce the largest difference between placebo-treated and drug-treated patients while retaining an acceptably low proportion of improved placebo-treated patients. We will follow the methodology outlined by Harold Paulus in previous work for RA. The index will be tested by applying it to the remaining set of study subjects (validation set) used to derive the criterion. Performance measures will include discriminative ability, calibration and overall accuracy.

## Conclusions

This new composite index will be simple to use, based on real individual patient clinical trial data, and will include patient-reported outcome measures. The index should serve to prevent useful drugs from being discarded due to inadequate trial designs. Preliminary data will be presented.

